# Effects of distraction methods on anxiety and pain during blood collection in children aged 9–13

**DOI:** 10.1007/s11845-025-04267-x

**Published:** 2026-01-13

**Authors:** Bahar Ürün Ünal, Burcu Gök Erdoğan, Fuat Buğrul, Hüseyin Can

**Affiliations:** 1 Department of Family Medicine, Selcuk University Faculty of Medicine, Konya, Turkey; 2 Department of Pediatric Endocrinology, Selcuk University Faculty of Medicine, Konya, Turkey; 3 Department of Family Medicine, Izmir Katip Celebi University Faculty of Medicine, Izmir, Turkey

**Keywords:** Child pain, Venipuncture procedure, Distraction, Buzzy, Cartoon

## Abstract

**Background:**

Non-pharmacological distraction methods are alternatives that can help relieve pain and anxiety during blood collection in pediatric patients.

**Aims:**

This study aimed to evaluate the effects of distraction methods used during blood collection on pain and anxiety in children aged 9–13. Wong-Baker FACES Pain Rating Scale (WB-FACES) and Visual Analog Scale (VAS) were used to assess pain and anxiety levels.

**Methods:**

A randomized controlled experimental study involving 450 pediatric patients aged 9–13 who applied to the pediatric endocrinology unit of a university hospital. During the blood collection procedure, children were randomly divided into 3 groups: watching cartoons, cold application (Buzzy), and control. Age-appropriate pain scales were used to assess pain.

**Results:**

The study included 450 children, 57.6% (*n* = 259) male and 42.4% (*n* = 191) female, with a mean age of 11.02 ± 1.45. One-third (*n* = 150) of the participants were shown cartoons, and one-third (*n* = 150) were given the cold application method. The remaining 1/3 (*n* = 150) were not given any distraction intervention during blood collection. Significantly lower pain scores were recorded in both intervention groups compared to the control group (*P* < 0.001 and *P* < 0.001).

**Conclusion:**

Less pain was noted in the Buzzy and cartoon groups compared to the control group. This suggests that these methods are effective non-pharmacological methods that can be used to reduce pain. The results show that these methods can be safely used for venipuncture pain in children aged 9–13 years.

## Introduction

According to the definition of the International Association for the Study of Pain (IASP), pain is an unpleasant sensation originating from any part of the body, accompanying existing or potential tissue damage or defined by this damage, encompassing all past experiences of a person [1]. The Joint Accreditation Commission of Healthcare Organizations recommends that pain be considered as the fifth vital sign [2]. Pediatric patients are frequently exposed to invasive procedures (intravenous catheterization, venipuncture and vaccination) that cause pain, anxiety, stress and fear during diagnosis and treatment [3]. These fears often lead to reluctance in children and parents to undergo medical procedures such as injections and blood sampling, affecting the child’s subsequent treatment and care experience. Children with chronic diseases in particular encounter many painful procedures during the diagnosis, treatment and follow-up process [4]. The study shows that children experience pain and anxiety during medical procedures [4]. The International Guide to Paediatric Anesthesia (Good Practice in Postoperative and Procedural Pain) recommends pharmacological and non-pharmacological methods for the effective management and prevention of acute procedural pain in children [5]. Non-pharmacological methods make it easier for children to cope with medical procedures and reduce anxiety and pain [6]. These methods are reliable and have no side effects [7]. By distracting the child, the child’s tolerance to pain can be increased by focusing on another stimulus. These techniques do not eliminate pain but help to make it more bearable [8]. Studies have evaluated a number of pharmacological and non-pharmacological interventions for the management of procedural pain in children. However, most of these interventions are not used by healthcare professionals because they are expensive, time-consuming or difficult to use [9]. Therefore, easy-to-use, practical, non-invasive, cost-effective and reusable non-pharmacological methods such as watching cartoons and Buzzy can be used, especially in acute situations. Studies directly comparing the Buzzy and cartoon methods are limited in the literature, which reveals the necessity of our study. The aim of this study was to evaluate the effects of cartoons and the Buzzy application in reducing pain and anxiety during venipuncture in children aged 9–13 years.

## Methods

### Study design

The study is a randomized controlled experimental trial conducted between August and November 2024 in the Pediatric Endocrinology Unit. 450 children aged 9–13 years who applied to Pediatric Endocrinology Unit were included in the study. Written informed consent was obtained from all participants and one of their parents before participating in the project. Since it was planned to include all children between the ages of 9 and 13 who applied to Pediatric Endocrinology Unit during the data collection process, volunteered, and signed an informed consent form by both themselves and their families, the targeted sample size or group could not be created. Children and/or parents who refused to participate in the study were not included in the study and the relevant data were not collected.

This study was approved on July 16, 2024 by our university Local Ethics Committee (Decision no. 2024/378). It was confirmed that all aims and instruments of the study were designed in accordance with the ethical standards of the institutional research committee, the Declaration of Helsinki and its subsequent amendments.

### Data collection

A questionnaire consisting of 18 questions was applied to the children by reviewing the literature and including data on age, gender, number of siblings, family structure, presence of chronic disease, history of surgery, age of parents, education level of parents, and employment status of parents. The data collected within the scope of this project was used for this study.

The children included in the study were those who were being followed up in the pediatric endocrinology department and required repeated blood collections for follow-up. Therefore, the first blood collection was performed under routine conditions and data were collected, and randomized distraction interventions were applied during the subsequent blood collection.

During the first application, no distraction was applied to the children during the blood collection process. A questionnaire form was applied.

In the second application, one of the distraction methods, watching cartoons and applying cold, was used. During the blood collection process, 1/3 of the children (*n* = 150) were randomly selected to watch cartoons and 1/3 (*n* = 150) were applied cold. The remaining 1/3 (*n* = 150) were considered as the control group and no intervention was applied during the blood collection process. Again, Wong Baker Pain Rating Scale, State-Trait Anxiety Inventory for Children, Visual Analog Scale (VAS) were applied in the second application. The answers given to the questionnaire for pain and anxiety assessments and the scores given to the scales were evaluated by the children.

A single blood sample was taken from the child patients included in the study using an 18-gauge needle by a permanent nurse. The nurse in the study worked between 08:00 and 16:00 and the blood sample was taken during the same time period.

### Scales and materials used

#### Wong-Baker FACES pain rating scale (WB-FACES)

**Fig. 1:**
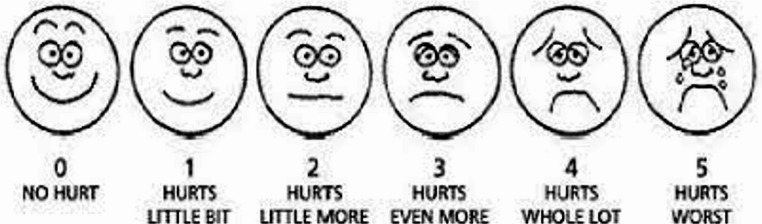
Wong-Baker FACES Pain Rating Scale (WB-FACES)

Wong-Baker FACES Pain Rating Scale (WB-FACES) was developed by Wong and Baker in 1981 and revised in 1983 [10] (Fig. 1). The scale is used to determine pain in children aged 3–18 years. It consists of six facial expressions representing increasing pain levels, scored from 0 to 5 from left to right (0 = very happy/no pain, 5 = worst pain imaginable). The first face is a happy face representing “no pain” (0), and the last face is a crying face representing “worst pain imaginable” (5). High scores indicate low pain tolerance. Participants are asked to choose the facial expression that best represents their pain. Wong Baker FACES Pain Assessment Scale was developed by Gerçeker et al. in 2020, Şahiner and Bal in 2016, and Şahiner et al. in 2015 in Turkiye [11–13].

#### State-Trait anxiety inventory for children

The scale was developed by Spielberger in 1973 [14]. The Turkish validity and reliability study was carried out by Özusta in 1993 [15]. In the State Anxiety Scale, children are asked to evaluate how they feel “at that moment” and to mark one of the three relevant options. The scale, consisting of twenty items, aims to evaluate feelings related to state anxiety such as tension, nervousness, and uneasiness. If the presence of these feelings is reported by the child as a lot, the highest score is 3, and if it is reported as not present, the lowest score is 1. The highest score that can be obtained from the State Anxiety Scale is 60, and the lowest score is 20. The total score is used in the evaluation of the scale (An increase in the score indicates a high level of anxiety). In the Turkish validity and reliability study of the scale, Cronbach’s Alpha coefficient was found to be 82. For the sample used in this study, Cronbach’s Alpha coefficient of the scale was found to be 81. Questions 2,4,5,7,9,11,15,16,18,19 were reverse coded.

#### Visual analog scale (VAS)

**Fig. 2:**
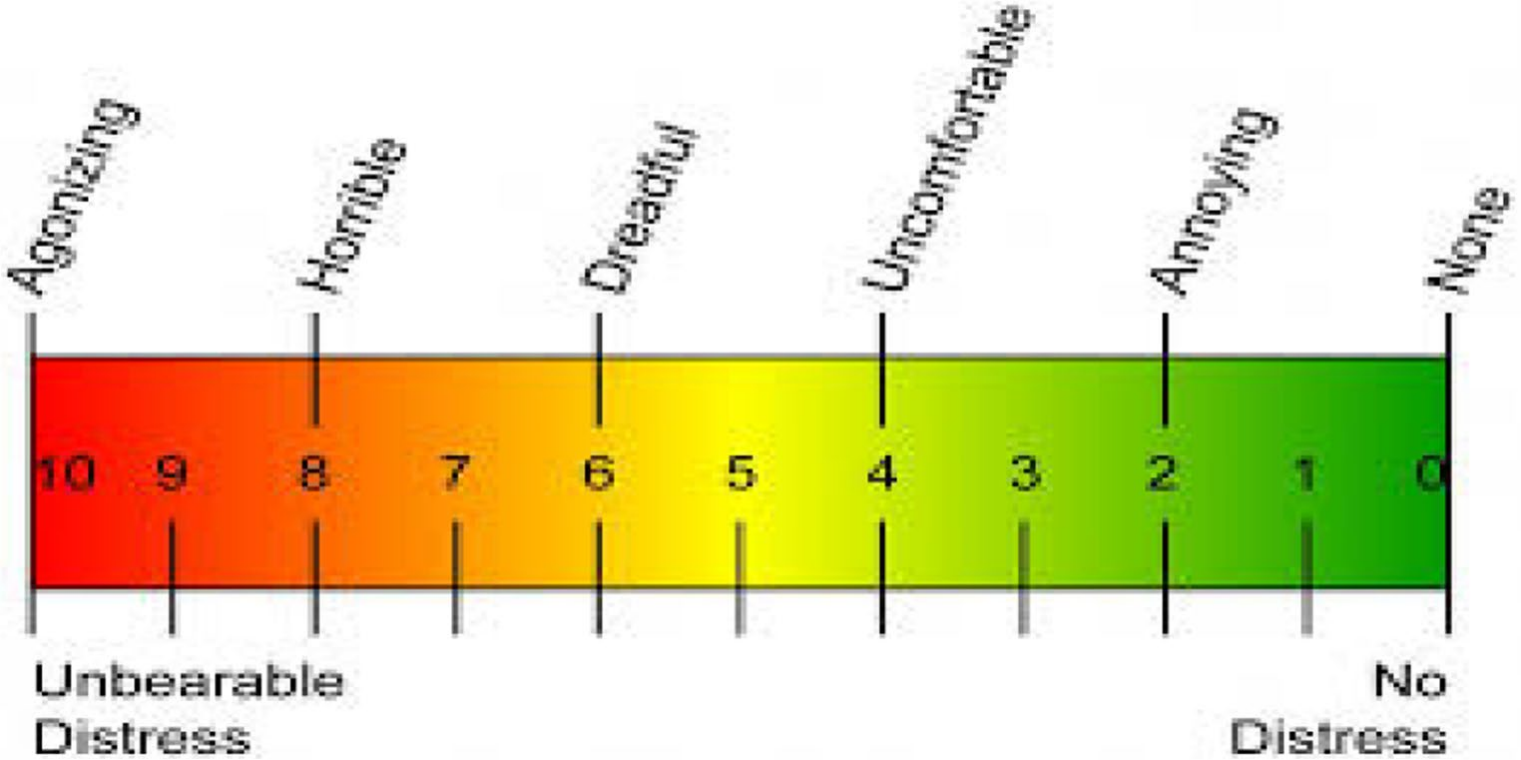
Visual Analog Scale (VAS)

The scale consists of a 10 cm body. 0 line indicates “no pain” 10 lines indicate “unbearable pain” [16] (Fig. 2). The location marked by the child indicates the severity of the pain [16]. The pain score is calculated by measuring the location of the mark with a ruler [16]. VAS is an easy-to-understand and easy-to-apply measurement tool.

#### Buzzy

Buzzy (MMJ Labs, Atlanta, GE, USA) is an easy-to-use, reusable, bee-shaped device consisting of a stem and wings designed to reduce injection pain in children [9]. The stem vibrates, while the wings apply intense cold to the injection site before injection [9]. The device should be placed approximately 5 cm above the puncture point for a few minutes before the procedure. The device helps relieve pain by emitting cold, which acts on the A-Delta fibers, and small vibrations, which act on the C-fibers [17].

#### Cartoons

The children were asked which cartoon they wanted to watch before the procedure. When the blood collection process was completed, the cartoon watching intervention was also terminated. Children in the cartoon group were shown a popular cartoon (a funny short animated film) appropriate for their age, starting just before the blood collection procedure and continuing throughout the procedure. The video was shown on the tablet screen.

#### Statistical analysis

Descriptive data were presented as number and percentage for categorical variables, and mean and standard deviation for continuous variables. The Chi-square test was used to evaluate the distribution of categorical variables between groups. The normality of continuous variables was assessed using the Kolmogorov–Smirnov test. Since the data did not show normal distribution, the Kruskal–Wallis test was applied for comparisons among more than two independent groups. When pairwise comparisons between independent groups were needed, the Mann–Whitney U test was used. For within-group comparisons of two dependent measurements, the Wilcoxon Signed-Rank test was performed. Statistical analyses were conducted using the SPSS 22 package program, and a significance level of *p* < 0.05 was adopted.

## Results

450 children (191 (42.4%) girls and 259 (57.6%) boys) were included in the study. The mean age of the children was 11.03 ± 1.45 years. The children were randomly divided into cartoon watching (*n* = 150), Buzzy (*n* = 150) and control (*n* = 150) groups. Table 1 presents the sociodemographic data of children and families and the data on scale assessments at the first application. There was no significant difference between the groups in terms of children’s age (*p* = 0.545), gender (*p* = 0.754), family structure (*p* = 0.417), mother’s education status (*p* = 0.935), father’s education status (*p* = 0.051), mother’s age (*p* = 0.836), father’s age (*p* = 0.716), mother’s employment status (*p* = 0.070), father’s employment status (*p* = 0.976), pre-procedure State-Trait Anxiety Inventory for Children score (*p* = 0.282), WB-FACES (*p* = 0.713), VAS (*p* = 0.955) scale scores. All children stated that they felt fear during previous blood collection procedures.

**Table 1:** Sociodemographic characteristics of children and scale scores before the first venipuncture

Characteristics		Cartoon group		Buzzy group		Control group		*p*
		*n*	%	*n*	%	*n*	%	
Gender*	Female	65	34.0	60	31.4	66	34.6	*0.754*
	Male	85	32.8	90	34.7	84	32.4	
Family Structure*	Small Family	129	34.7	120	32.3	123	33.1	*0.417*
	Large Family	21	27.3	30	39.0	26	33.8	
	Divorced Parents	0	0.0	0	0.0	1	100.0	
		**Mean ± SD**		**Mean ± SD**		**Mean ± SD**		
Age (year)**		10.94 ± 1.43		11.04 ± 1.51		11.16 ± 1.52		*0.545*
Mother’s Age (year)**		37.24 ± 6.00		37.78 ± 6.24		37.64 ± 6.23		*0.836*
Father’s Age (year)**		38.63 ± 6.08		38.24 ± 6.48		39.61 ± 6.23		*0.716*
WB-FACES (score)**		3.82 ± 0.07		3.98 ± 0.07		4.04 ± 0.067		*0.713*
VAS (score)**		7.55 ± 0.13		7.42 ± 0.13		7.52 ± 0.13		*0.955*
State-Trait Anxiety Inventory for Children (score)**		51.80 ± 2.31		51.07 ± 2.75		52.01 ± 2.59		*0.282*

*Chi-square test, ** Kruskal-Wallis test

Both the cartoon and Buzzy groups had significantly lower wong and vas scores than the control group (*P* < 0.001 and *P* < 0.001). There was no significant difference in the State-Trait Anxiety Inventory for Children scores between the groups (*p* = 0.965) (Table 2).

**Table 2:** Comparison of pain levels felt by children during the 2nd venipuncture according to groups

Characteristics	Cartoon group	Buzzy group	Control group	*p*
	Mean ± SD	Mean ± SD	Mean ± SD	
WB-FACES (score)	1.54 ± 0.05	1.65 ± 0.05	3.97 ± 0.07	*P* < 0.001
VAS (score)	3.10 ± 0.08	3.51 ± 0.07	7.41 ± 0.13	*P* < 0.001
State-Trait Anxiety Inventory for Children (score)	53.29 ± 2.04	53.53 ± 1.86	53.05 ± 2.13	*0.965*

### Kruskal-Wallis test

Both groups (Buzzy and cartoon) that received the distraction intervention had similarly lower VAS and WB-FACES scores than the control group.

Children’s WB-FACES and VAS scores at the first application were significantly higher than their WB-FACES and VAS scores at the second application (*P* < 0.001 and *P* < 0.001) (Table 3).

**Table 3:** Comparison of children’s WB-FACES and VAS scores at the first and second applications

	*n*	Mean ± SD	*p*
WB-FACES (1) WB-FACES (2)	450 450	4.00 ± 0.04 2.39 ± 0.06	***P*** **< 0.001**
VAS (1) VAS (2)	450 450	7.50 ± 0.07 4.67 ± 0.10	***P*** **< 0.001**

### Paired samples t-test

The State-Trait Anxiety Inventory score of the children at the first application was 51.62 ± 2.59, and at the second application it was 53.29 ± 2.02. No significant difference was found between the first and second inventory scores (*p* = 0.052).

## Discussion

In the literature review, we did not come across another study that divided into 3 groups as Buzzy, cartoon watching and control group as in our study. Our study has important results because it is a rare study comparing these two attention-grabbing methods in terms of ease of use and access.

Blood collection is a routine medical procedure performed in hospitals and clinics and can not only cause pain in children but also lead to fear, stress and anxiety [18]. Although it is possible that the type of procedure the child undergoes is related to the level of pain and anxiety felt during the procedure, the emotional state of the child and his/her parents, previous similar experiences, and the performance of the physicians are also among the individual factors affecting the outcome [18, 19]. In order to control acquired pain and the resulting future anxiety behavior, not only pharmacological but also non-pharmacological measures should be used [20].

In our study, it was determined that the demographic characteristics and scale scores of the children were similar. The fact that there was no statistical difference in terms of both demographic characteristics and pre-intervention State-Trait Anxiety Inventory for Children, WB-FACES and VAS scores of the children is an indication of successful randomization. Thus, it can be stated that there was a homogeneous distribution between the groups in terms of factors triggering pain, anxiety and fear. Similar to our study, in a study examining the effects of attention-drawing interventions conducted with children in 2022 on venipuncture pain and anxiety, it was shown that the groups were similar in terms of clinical and demographic characteristics, and this was stated to be an indication of successful randomization [21].

In literature reviews, there are studies examining the performance of various non-pharmacological methods (cold application, watching cartoons, virtual reality glasses, distraction cards, use of kaleidoscope, etc.) on venipuncture pain and anxiety in children [9, 22–26].

In our study, the effects of two non-pharmacological methods (showing cartoons and Buzzy) on venipuncture pain and anxiety in children aged 9–13 years were investigated. The results show that both methods help reduce venipuncture pain and anxiety in children. In a study conducted in 2020, children aged 6–12 years were divided into four groups (control, Buzzy, distraction cards, and Buzzy + distraction cards groups) and the effects of distraction methods on venipuncture pain were examined, and it was reported that the distraction cards and Buzzy + distraction card groups had lower pain scores than controls [27]. Similarly, in a study conducted in 2018, which investigated the effects of non-pharmacological methods (Buzzy, showing cartoons, Buzzy + watching cartoons) on venipuncture pain management in children aged 5–12 years, it was stated that all of these non-pharmacological methods were effective on venipuncture pain and anxiety in children [23]. Another study reported that using Buzzy during and after phlebotomy in children aged 5–10 years was effective in reducing pain and reducing children’s fear levels [25]. In a meta-analysis study conducted by Ballard et al. in 2019, they examined the effectiveness of Buzzy, which combines cold and vibration for needle-related procedural pain in children and reported that Buzzy is an effective intervention for procedural pain management in children [9]. Our results, in line with the literature, suggest that Buzzy can be used as an effective method in venipuncture.

Cartoons and animated videos are also a distraction used to distract children from venipuncture pain and anxiety [28]. Cartoons and animated videos are relatively simple and quick methods to distract children and prepare them for invasive procedures [28]. In our study, it was found that watching cartoons was statistically significantly effective in eliminating venipuncture pain. Similar to our results, Düzkaya et al. reported in 2021 that watching animated videos and cartoons was effective in reducing pain caused by peripheral intravenous catheterization in children aged 6–12 years [28]. In a similar study conducted with outpatients in the pediatric department in Italy, it was stated that there was a statistically significant difference in children’s pain perception in the group that watched cartoons during the venipuncture procedure [29]. Another study conducted in Turkiye reported lower anxiety scores in the group that watched cartoons during the procedure [28].

In our study, although no statistically significant difference was found between the anxiety and fear scores of the cartoon watching group and the Buzzy group during venipuncture, the VAS and WB-FACES scale scores were lower in the cartoon group. There are many studies in the literature examining attention-drawing interventions for venipuncture pain and anxiety in children. However, studies comparing the Buzzy and cartoon watching methods, which are perhaps the most applicable and easy to access among these interventions, are limited. In the study by Bergomi et al. published in 2018, it was reported that the group receiving the distraction intervention with animated cartoons was the most effective method in terms of reducing pain perception in children when compared to the Buzzy group [29]. No significant relationship was found between gender and venipuncture pain and anxiety in the study. Similar to our results, many studies have reported that gender does not affect pain and anxiety. However, there are also results in the literature showing that male gender indicates higher pain tolerance [30–32].

Less pain was recorded in the Buzzy and cartoon groups compared to the control group. Our study showed the effectiveness of these non-pharmacological methods in pain control during venepuncture.

The results show that watching Buzzy and cartoons are effective non-pharmacological methods that can be used to reduce pain. It also shows that these methods can be used safely for venepuncture pain in children aged 9–13.

It is important to provide methods that will be integrated into painful interventions in medicine. In addition, there is a need for studies that appeal to larger and broader audiences with non-pharmacological distraction methods that can be applied during blood collection.

There are several limitations to the study. First, the study was not double-blind. Children may have responded in ways they felt were expected to. It is also possible that children may have been confused about the distinction between fear and anxiety during painful procedures.

## Data Availability

The datasets generated and/or analyzed during the current study are not publicly available due to ethical restrictions and the protection of participant confidentiality, but are available from the corresponding author on reasonable request.
